# MiR‐9 promotes angiogenesis of endothelial progenitor cell to facilitate thrombi recanalization via targeting TRPM7 through PI3K/Akt/autophagy pathway

**DOI:** 10.1111/jcmm.15124

**Published:** 2020-03-09

**Authors:** Dong‐Ming Zhou, Li‐Li Sun, Jian Zhu, Bing Chen, Xiao‐Qiang Li, Wen‐Dong Li

**Affiliations:** ^1^ Department of Hematology The Affiliated Drum Tower Hospital Nanjing University Medical School Nanjing China; ^2^ Department of Vascular Surgery The Affiliated Drum Tower Hospital Nanjing University Medical School Nanjing China; ^3^ Department of Vascular Surgery The Second Affiliated Hospital of Soochow University Suzhou China

**Keywords:** angiogenesis, endothelial progenitor cells, migration, miR‐9‐5p, thrombosis, TRPM7

## Abstract

Endothelial progenitor cells (EPCs) have emerged as a promising therapeutic choice for thrombi recanalization. However, this role of EPCs is confined by some detrimental factors. The aim of this study was to explore the role of the miR‐9‐5p in regulation of the proliferation, migration and angiogenesis of EPCs and the subsequent therapeutic role in thrombosis event. Wound healing, transwell assay, tube formation assay and in vivo angiogenesis assay were carried out to measure cell migration, invasion and angiogenic abilities, respectively. Western blot was performed to elucidate the relationship between miR‐9‐5p and TRPM7 in the autophagy pathway. It was found that miR‐9‐5p could promote migration, invasion and angiogenesis of EPCs by attenuating TRPM7 expression via activating PI3K/Akt/autophagy pathway. In conclusion, miR‐9‐5p, targets TRPM7 via the PI3K/Ak/autophagy pathway, thereby mediating cell proliferation, migration and angiogenesis in EPCs. Acting as a potential therapeutic target, miR‐9‐5p may play an important role in the prognosis of DVT.

## 1 INTRODUCTION

Deep vein thrombosis (DVT) and its chronic sequelae have a profound impact on health‐related quality of life.^1^ However, currently, there are no specific therapeutic strategies to avoid its complications, especially post‐thrombotic syndrome (PTS).^2^, ^3^, ^4^ Endothelial progenitor cells (EPCs), which can accelerate thrombus recanalization by restoring damaged or lost endothelium, enhancing neovascularization and prompting thrombi resolution, have been described as a potential therapeutic application for thrombosis.^5^, ^6^, ^7^ But the low numbers of EPCs in peripheral blood and the features of fragility to detrimental homeostasis limit their role in the resolution of thrombi. Therefore, there is an urgent need for seeking a practical, valid and reliable method to promote the proliferation and angiogenesis of EPCs.

MicroNRAs are short non‐coding RNAs that have been identified with crucial roles in venous thrombosis.^8^, ^9^, ^10^ Besides, microRNAs play an important role in the modulation of stem cells and angiogenesis.^11^, ^12^, ^13^, ^14^ The survival, mobilization and angiogenesis of EPCs also can be regulated by microRNAs, which facilitate the recanalization of thrombi and may exhibit a potential therapeutic application for DVT.^12^, ^13^ It was demonstrated that miR‐9‐5p played an important role in migration, metastasis and angiogenesis of cells.^15^, ^16^, ^17^ But its role in the migration and angiogenesis of EPCs remains unclear.

Here, we demonstrated miR‐9‐5p could promote the proliferation, migration and angiogenesis of EPCs. Moreover, the role of miR‐9‐5p in the process of venous thrombus resolution in thrombosis model showed a potential therapeutic intervention for EPC‐mediated angiogenesis in DVT.

## 2 MATERIALS AND METHODS

### 2.1 Ethical approval of the study protocol

All researches involving human participants were approved by the Institutional Review Board of the Nanjing Drum Tower Hospital Affiliated to Medical School of Nanjing University, Nanjing, China, and written informed consent was obtained from each participant. The study protocol was approved by the Institutional Animal Care and Use Committee of Nanjing University. All the animal experimentations were conducted following international guidelines.

### 2.2 Isolation and characterization of human EPCs

Endothelial progenitor cells were isolated and characterized as previously described.^18^, ^19^ Peripheral blood mononuclear cells, which were isolated using a Ficoll‐Isopaque Plus (Histopaque‐1077; Sigma) gradient centrifugation method, were seeded in fibronectin‐coated cell culture flasks. Then, the cells were cultured with Endothelial Basal Medium‐2 (EBM‐2; Lonza) in a 37°C, 5% CO_2_ incubator. Culture media were replaced after 4 days of incubation. Late EPCs grow into cobble‐stone‐like colonies after 14 days of incubation. The presence of EPCs was validated by both confocal microscopy and flow cytometry as previously described.^20^, ^21^ EPCs were identified by double‐positive for DiI‐Ac‐LDL and UEA‐1. Surface makers, including CD31, CD34, CD45, CD133 and CD309 (Becton‐Dickinson), were analysed for the definition of EPCs. The EPCs from 3 to 5 passages were used.

### 2.3 Cell treatments

Recombinant lentiviral particles carrying miR‐9‐5p mimics and inhibitor and vehicle controls were provided by GenePharm Co. Ltd. Endothelial progenitor cells were infected according to previous methods.^20^ Briefly, cells were infected with the above lentiviral particles for 48 hours when cells were grown to approximately 40% confluence. Cationic polymer polybrene (8 μg/mL in water) was used to enhance the infection efficiency.

### 2.4 Cell counting kit (CCK)‐8 assay for cell growth

Endothelial progenitor cells (3000 cells) were seeded in 96‐well plates, and then incubated in complete EBM‐2 medium overnight at 37°C, 5% CO_2_ for adhesion. Cell proliferation was evaluated at 0, 24, 48, 72 and 96 hours using a CCK‐8 assay referring to the manufacturer's instructions. The absorbance at 450 nm was detected by microplate reader (Molecular Devices). All the tests were performed at least in triplicate.

### 2.5 Apoptosis analysis by flow cytometry

Endothelial progenitor cells were stained with APC Annexin V Apoptosis Detection Kit with 7‐AAD (BioLegend) according to the manufacturer's instructions, and analysed by flow cytometry.

### 2.6 Wound healing

Endothelial progenitor cells were allowed to be grown to 80%‐90% cell confluence. A scratch was made by 200 µL pipette tip at 0 hour time‐point and incubated 37°C, 5% CO_2_. Pictures were taken under a microscopy at 0 and 24 hours for migration evaluation.

### 2.7 Transwell assay

A modified transwell assay with 8 μm pore filter inserts in 24‐well plates was used for cell migration evaluation (BD Biosciences). After cell transfection for 24 hours, cells were suspended with serum‐free at the concentration of 3 × 10^5^ cells and added to the upper chamber, while lower chamber was filled with EGM‐2 supplemented with 20% FBS as a chemoattractant. Then, the transwell plate was incubated at 37°C for 24 hours, and a cotton swab was used for upper chamber residue cells removing. The transwell membrane close to the lower chamber was cut by a scissor, stained with crystal violet and photographed by a microscope. The cells were finally counted according to the pictures.

### 2.8 Tube formation assay

An in vitro Matrigel tube formation assay was performed as described previously.^20^ Briefly, EPCs (1 × 10^4^ cells per 100 µL medium) were infected with miR‐9‐5p mimic or inhibitor lentivirus and then seeded in Matrigel‐coated 96‐well plates. Tube structures were inspected under an inverted light microscope after 12 hours of incubation. The total number of tubes was scanned and quantified in three random fields per well.

### 2.9 In vivo angiogenesis

The capacity of angiogenesis in EPCs was also assessed by in vivo tube formation assay as described previously.^20^, ^22^, ^23^ Six‐week‐old male athymic nude (nu/nu) mice were purchased from Shanghai SLAC Laboratory Animal Co. Ltd. Cells (5 × 10^5^ cells) that were infected with miR‐9‐5p mimics, siRNA lentivirus or vehicle controls were resuspended in 200 μL of Matrigel (BD) on ice and injected subcutaneously into the flank of the mice (n = 6). Seven days later, the Matrigel implants were harvested, fixed in 4% paraformaldehyde, embedded in paraffin. The histological sections were stained with haematoxylin and eosin (H&E). Luminal structures that contained erythrocytes were considered as microvessels. Four randomly selected fields in each section were counted under 200× magnification.

### 2.10 Luciferase assay

Luciferase reporter assay was performed as previously described to explore the potential regulation mechanisms of miR‐9‐5p.^20^ Cells were cotransfected with firefly luciferase reporter plasmid (TRPM7‐WT, TRPM7‐MUT) and a Renilla luciferase vector (Promega) inserted with NC or miR‐9‐5p mimics. The activities of luciferase and Renilla plasmid were measured 48 hours later via the Dual‐Luciferase Reporter 1000 Assay System (Promega).

### 2.11 Reverse transcription‐quantitative PCR (RT‐qPCR)

Total RNA from EPCs was extracted using TRIzol Reagent in accordance with the manufacturer's instructions. cDNA for RT‐qPCR was synthesized using a Qiagen miRNA first‐strand synthesis and qPCR kit (miScript II; Qiagen) in accordance with the manufacturer's instructions. Real‐time PCR analysis was performed using an Mx3000P qPCR system (Agilent) with an miScript SYBR Green PCR Kit (Qiagen). Real‐time PCR conditions were as follows: 95°C for 15 minutes, followed by 40 cycles of 94°C for 15 seconds, 55°C for 30 seconds and 70°C for 30 seconds. Melting curve analysis was applied to analyse the PCR products. Relative miRNA expression was normalized to the reference miRNA expression performed with the ΔΔCt method as previously described.^24^ The oligonucleotide primers for miR‐9‐5p, TRPM7, GAPDH and U6 snRNA are listed in Table 1.

**Table 1:** Primer sequence

Gene name	Sequence
MiR‐9‐5p	5′‐UCUUUGGUUAUCUAGCUGUAUGA‐3′
U6	5′‐CGCTTCACGAATTTGCGTGTCAT‐3′
TRPM7	
Forward primer	5′‐ACTGGAGGAGTAAACACAGGT‐3′
Reverse primer	5′‐TGGAGCTATTCCGATAGTGCAA‐3′
GAPDF	
Forward primer	5′‐CATGAGAAGTATGACAACAGCCT‐3′
Reverse primer	5′‐AGTCCTTCCACGATACCAAAGT‐3′

### 2.12 Western blot analysis

Endothelial progenitor cells (1 × 10^5^ cells) were lysed in RIPA buffer, followed by high‐speed centrifugation and quantification using bicinchoninic acid. Cellular proteins were separated using sodium dodecyl sulphate‐polyacrylamide gel electrophoresis and transferred onto polyvinylidene difluoride membranes. After blocking, membranes were incubated with antibodies against TRPM7, phospho‐PI3K, PI3K, phospho‐Akt, Akt, LC3B and p62 (Cell Signaling Technology). Actin (Sigma) was applied as the loading control. Appropriate horseradish peroxidase‐conjugated secondary antibodies were used. Protein bands were detected with SuperSignal West Pico Chemiluminescent Substrate (Pierce) on X‐ray films (Kodak).

### 2.13 Mice model venous thrombosis

Male nude mice (10 weeks old) were anaesthetized, and the left jugular vein was isolated and ligated at the proximal end of the heart. miR‐9‐5p mimics EPCs and NC EPCs (5 × 10^6^) were injected into the thrombus. After 7 days, thrombi with vessel walls were harvested for measurements of size and weight, and for immunofluorescence.

### 2.14 Immunofluorescence analysis

Endothelial progenitor cells were fixed with 4% paraformaldehyde and blocked with Immunol Staining Blocking Buffer (Beyotime, P0102). Immunostaining was performed using TRITC Phalloidin (Solarbio, CA1610, 1:2000) to identify F‐actin. For immunostaining of CD31, frozen sections of thrombus were stained with anti‐CD31 (abcam; ab24590, 1:100). After incubation with Cy3‐labelled secondary antibodies (abcam; ab6939, 1:500), images were acquired using a confocal microscope under the same conditions for each experiment.

### 2.15 Statistical analysis

SPSS v21 (SPSS, Chicago, IL) was used to deal with all statistical analysis. Data are listed as mean ± standard deviation. A Student's *t* test or one‐way ANOVA was applied to analyse the differences between groups. *P* < .05 was considered statistically significant.

## 3 RESULTS

### 3.1 MiR‐9‐5p promoted the proliferation but inhibited the apoptosis of EPCs

**Figure 1:**
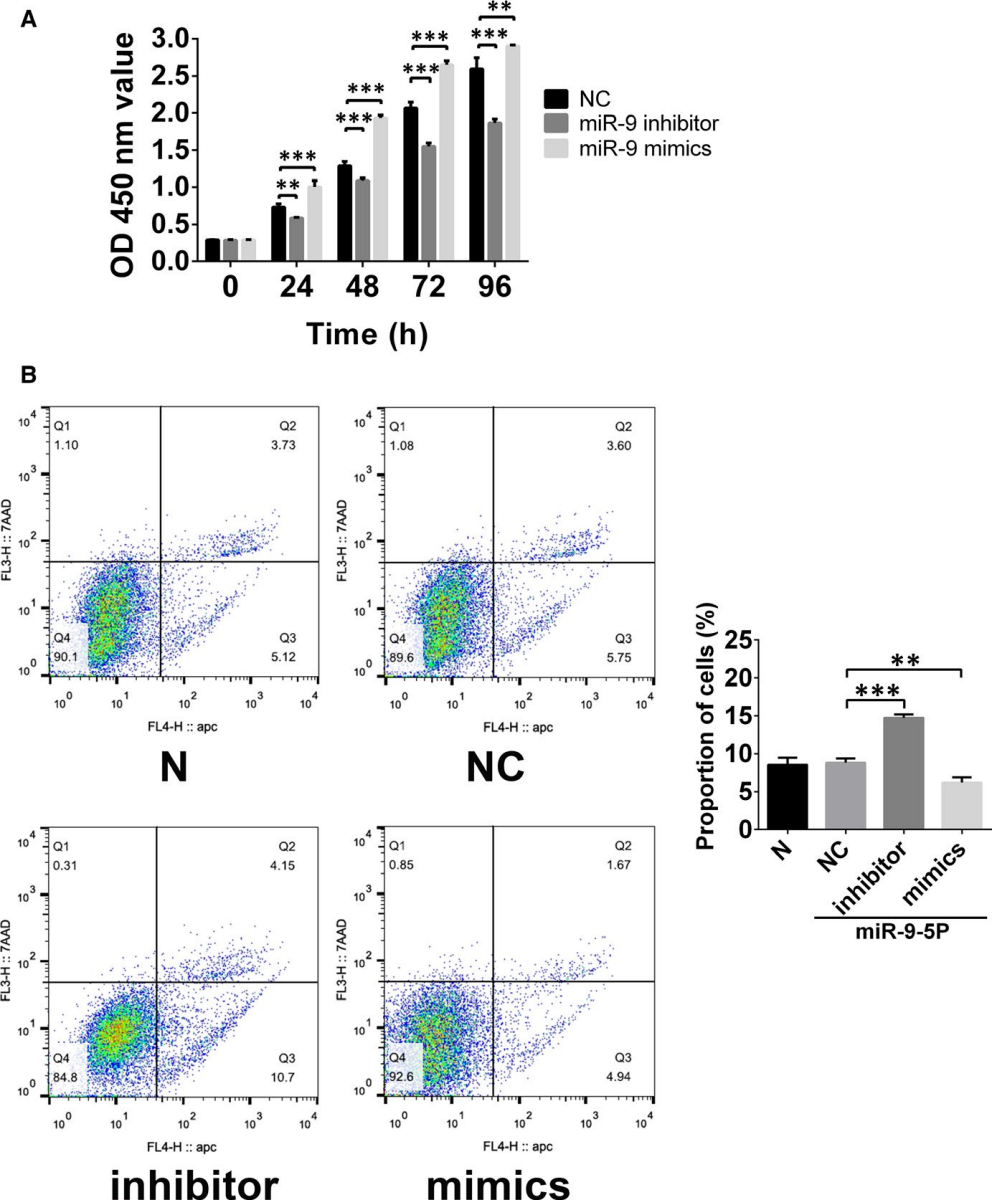
The role of miR‐9 in the regulation of endothelial progenitor cells (EPCs) growth. A, Cell proliferation was assessed by the Cell Counting Kit‐8 (CCK‐8) assay. The results showed that miR‐9 could promote the proliferation of EPCs. B, Apoptosis of EPCs regulated by miR‐9 was assessed by APC Annexin V Apoptosis Detection Kit. The results showed that miR‐9 could inhibit the apoptosis of the cells. **P < .01 and ***P < .001 for between‐group comparisons

Cells cultured in the study matched with the previously described EPC phenotype. These cells were positive staining of CD309 and CD31, weak positive staining of CD34, but negative staining of CD45 and CD133. To study the influence of miR‐9‐5p on EPCs growth, CCK‐8 assay and apoptosis analysis were performed. As shown in Figure 1, miR‐9‐5p inhibitor could attenuate the proliferation of EPCs (Figure 1A) and promote the apoptosis of EPCs (Figure 1B). While miR‐9‐5p mimics exhibited a contrary result.

### 3.2 MiR‐9‐5p promotes EPCs migration and tube formation

**Figure 2:**
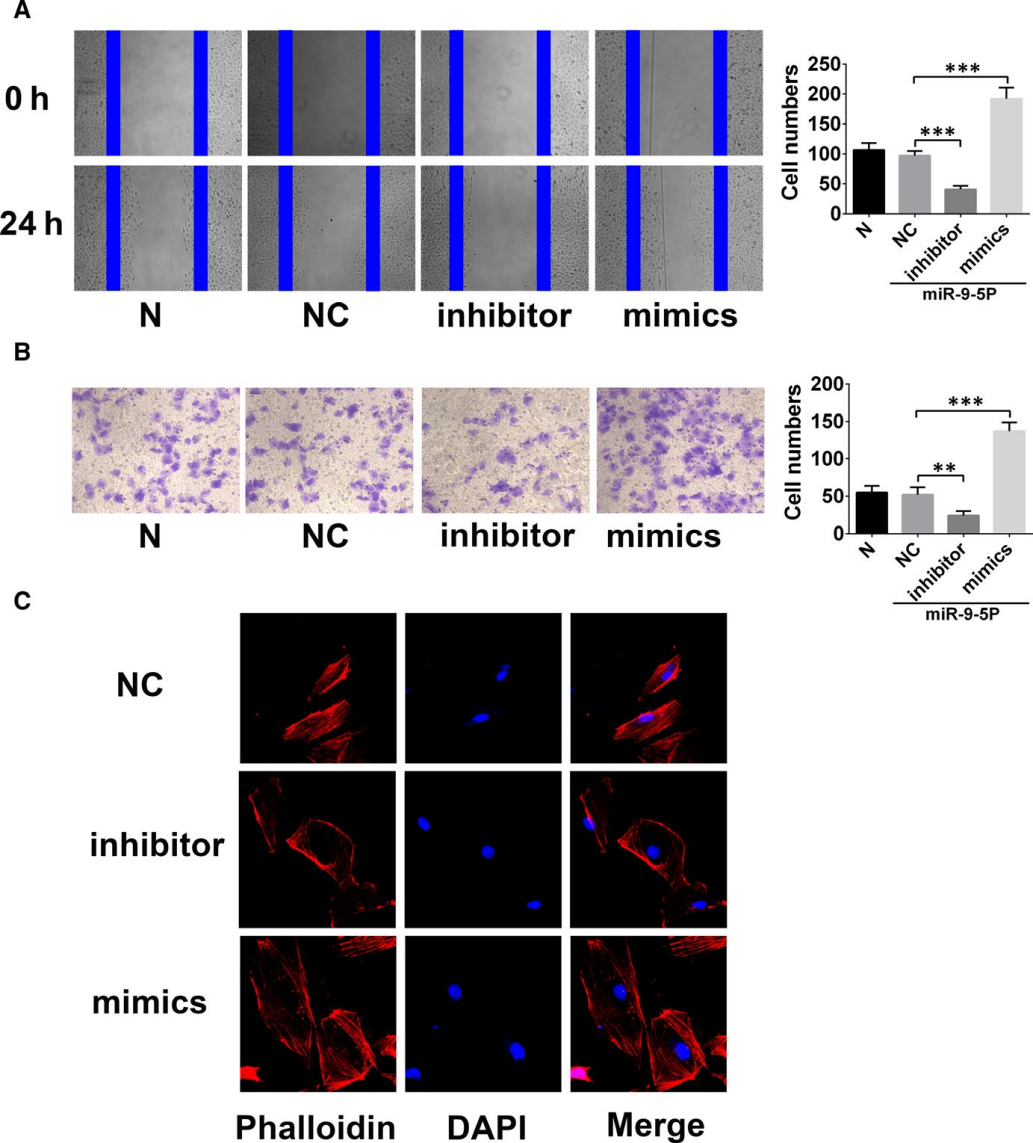
miR‐9‐5p promotes the migration and invasion of endothelial progenitor cells (EPCs). A, Wound healing assay showing the effects of miR‐9‐5p on EPC migration. miR‐9‐5p inhibitor and mimics, respectively, decreased and increased cell migration in EPCs (magnification, ×100). B, Transwell cell invasion assay provided results similar to those for wound healing (magnification, ×200). C, Effect of miR‐9‐5p on F‐actin in EPCs. Cells were stained with rhodamine‐phalloidin and visualized by confocal microscopy; miR‐9‐5p inhibitor impaired F‐actin filaments but miR‐9‐5p mimics prevented disruption of F‐actin filaments (magnification, ×400). **P < .01 and ***P < .001 for between‐group comparisons

**Figure 3:**
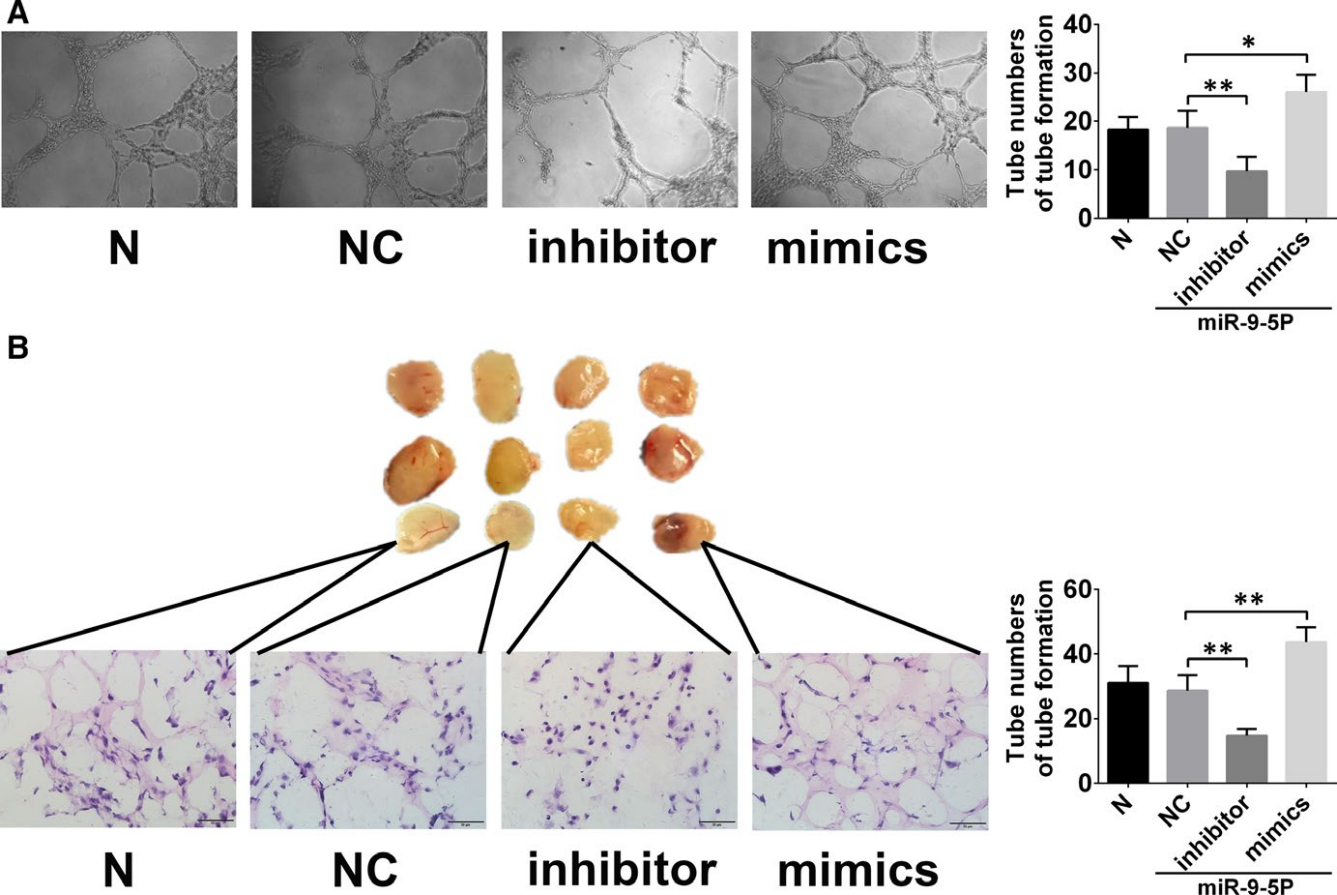
miR‐9‐5p promotes angiogenesis of endothelial progenitor cells (EPCs). A, In vitro tube formation assay. miR‐9‐5p inhibitor and mimics, respectively, decreased and increased tube formation by EPCs in vitro (magnification, ×100). B, In vivo tube formation was evaluated at Day 7 after subcutaneous injection of Matrigel‐mixed EPCs into nude mice. miR‐9‐5p inhibitor and mimics, respectively, decreased and increased tube formation by EPCs in vivo. *P < .05, **P < .01 and ***P < .001 for between‐group comparisons

To validate the role of miR‐9‐5p in EPCs, scratch assay and transwell assay were performed to evaluate the regulation of migration. As shown in Figure 2, miR‐9‐5p could positively regulate the migration of EPCs (Figure 2A,B). Besides, miR‐9‐5p mimics could enhance the expression of microfilament protein with the immunofluorescence staining by phalloidin. In vitro and in vivo tube formations were performed to assess the role of angiogenesis regulated by miR‐9‐5p. Both of the two assays displayed that miR‐9‐5p mimics could enhance the tube numbers. These results suggested that enhanced miR‐9‐5p could promote cell migration and angiogenesis of EPCs (Figure 3A,B).

### 3.3 TRPM7 is the target gene of miR‑9‑5p in EPCs

**Figure 4:**
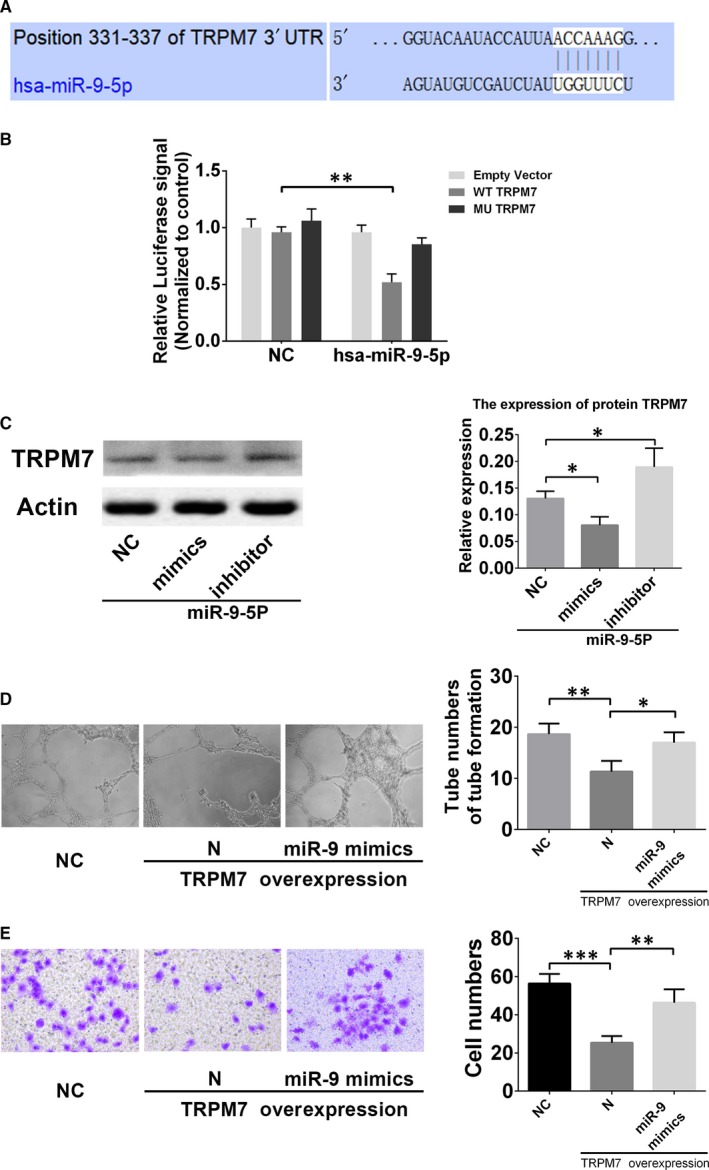
TRPM7 is a validated target of miR‐9‐5p. A, Putative binding sites of miR‐9‐5p in the TRPM7 3′UTR predicted by TargetScan. B, Dual‐luciferase reporter assay verified the targeting relationship between miR‐9‐5p and TRPM7. C, Western blot revealed that miR‐9‐5p mimics and inhibitor, respectively, decreased and increased protein expression of TRPM7 in EPCs. D and E, In vitro tube formation assay and transwell cell invasion assay showed that miR‐9‐5p mimics could reverse the inhibition of tube formation and invasion of EPCs by TRPM7 overexpression. (magnification, ×100 & ×200). *P < .05 and **P < .01 for between‐group comparisons

TRPM7 was predicted to be one of the targets of miR‐9‐5p via the TargetScan databases (Figure 4A). To confirm whether TRPM7 were regulated by miR‐9‐5p, luciferase reporter assays were performed, finding that miR‐9‐5p mimics could robustly reduce the group cotransfected with the WT TRPM7 plasmid (Figure 4B). These results confirmed that miR‐9‐5p interacted with the 3′‐UTR segment of TRPM7. To validate the ability of miR‐9‐5p to suppress the expression of TRPM7, miR‐9‐5p mimics and inhibitor were transfected into EPCs. These findings showed that miR‐9‐5p mimics exhibited significantly decreased TRPM7 protein level, while miR‐9‐5p inhibitor increased TRPM7 protein level in EPCs (Figure 4C).

To detect the role of TRPM7 in the regulation of EPCs migration and angiogenesis, transwell assay and in vitro tube formation were performed. As shown in Figure 4D,E, EPCs with TRPM7 knockdown by siRNA displayed a decreased cell migration, as well as the decreased numbers of tube formation.

### 3.4 The role of PI3K/Akt/autophagy pathway in regulation of miR‐9‐5p

**Figure 5:**
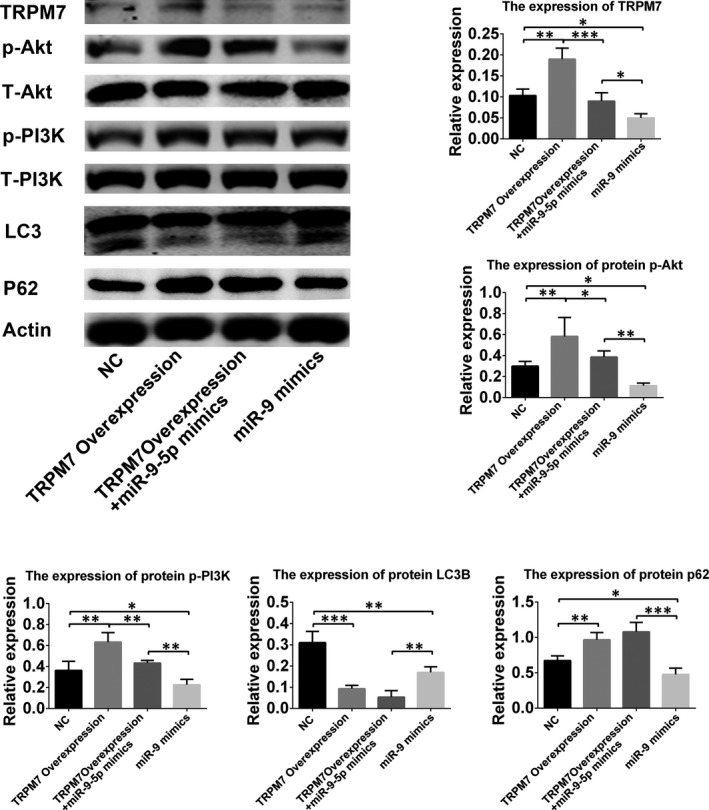
Involvement of PI3K/Akt/autophagy signalling pathway in miR‐9‐5p‐mediated effects in endothelial progenitor cells (EPCs). Western blot revealed that miR‐9‐5p mimics could inhibit the phosphorylation of Akt and PI3K. It could reverse the promotion of the phosphorylation of Akt and PI3K regulated by TRPM7 overexpression. MiR‐9‐5p could also reverse the expression of LC3B and p62 inhibited by TRPM7 overexpression. *P < .05, **P < .01 and ***P < .001 for between‐group comparisons

It has been demonstrated that PI3K/Akt/autophagy pathway is closely related to the regulation of cells migration and angiogenesis.^20^, ^25^, ^26^, ^27^ The results also indicated the down‐regulation and up‐regulation of PI3K and Akt phosphorylation were found in EPCs with miR‐9‐5p mimics and inhibitor, respectively (Figure 5). In addition, autophagy‐related proteins, including LC3B and P62, were found to be down‐regulated and up‐regulated, respectively, in EPCs with miR‐9‐5p inhibitor. These results confirmed that PI3K/Akt/autophagy pathway played an important role in the miR‐9‐5p mediated effects on EPCs.

### 3.5 MiR‐9‐5p promotes the angiogenesis of EPCs in thrombus and subsequent recanalization of thrombosis

**Figure 6:**
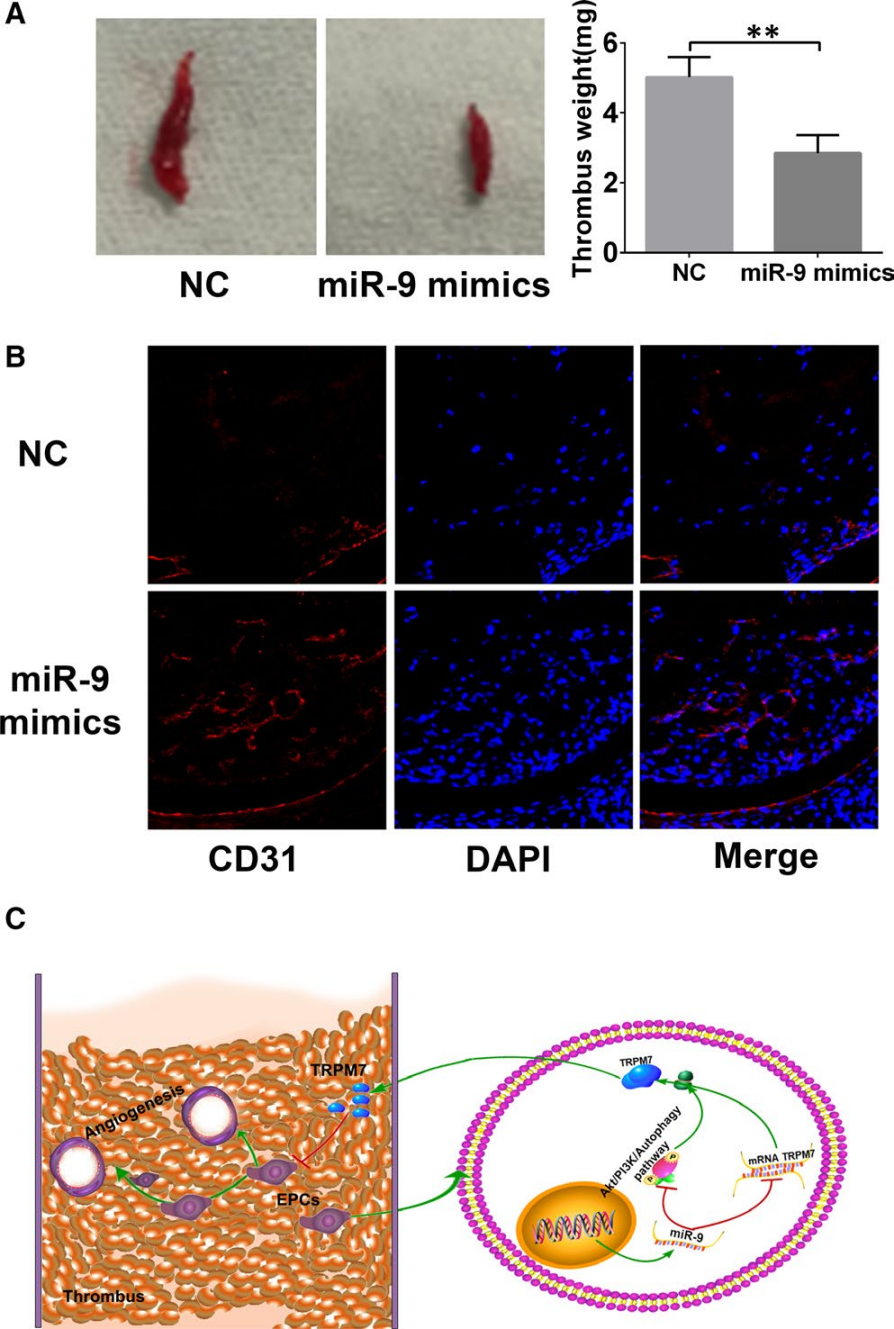
MiR‐9‐5p promotes endothelial progenitor cells (EPCs) homing and thrombus organization in vivo. A, Representative size and weight of thrombus after 7 d since EPCs were injected. Data are expressed as mean ± SD. **P < .01 for between‐group comparisons. B, The angiogenic capacity of EPCs in thrombus 7 d after injection of EPCs. MiR‐9‐5p could enhance the angiogenesis of EPCs in thrombus and subsequently promote thrombus resolution. C, Schematic of the role of miR‐9‐5p‐mediated effects in EPCs

Endothelial progenitor cells play an important role in the resolution of thrombi via promoting the angiogenesis and endothelium repairment.^5^ In this study, a model of DVT was established by ligation of a jugular vein and its branches. Endothelial progenitor cells transfected with NC or miR‐9‐5p mimics were intravenously administrated into nude mice. The results showed that, compared with NC, the thrombus size and the thrombus weight were significantly reduced in the group of EPCs infected with miR‐9‐5p mimics at day 7 after injection (*P* < .01, Figure 6A). In addition, immunostaining with CD31 showed capillary‐like structure in thrombus was more in the group of miR‐9‐5p mimics (Figure 6B). This demonstrated that miR‐9‐5p could promote the angiogenesis of EPCs in thrombus and subsequent recanalization of thrombosis.

## 4 DISCUSSION

In the present study, we investigated the effects of miR‐9‐5p on the angiogenic capacity of EPCs and the subsequent role in thrombus recanalization. It was found that miR‐9‐5p prevented apoptosis but promoted proliferation, migration and angiogenesis of EPCs, which enhanced the recanalization of venous thrombosis. This might be attributed to its targeted gene named TRPM7. And PI3K/Akt/autophagy pathway was involved into this process of regulation (Figure 6C).

It has been shown that miR‐9 could enhance endothelial cell (EC) migration and angiogenesis^28^ but inhibit EC apoptosis and inflammation.^17^ In addition, enhanced angiogenesis was found in ECs and bone marrow stromal cells (BMSCs), as well as in osteoblasts.^17^, ^29^, ^30^ In the present study, a consistent result was found in EPCs via up‐regulation of miR‐9. However, in some of the previous studies, it was also found to inhibit the proliferation of smooth muscle cells and BMSCs as the contrary results.^31^, ^32^ Besides, in nasopharyngeal carcinoma, exosome miR‐9 was found to inhibit angiogenesis.^33^ The different roles of miR‐9 in the regulation of cell proliferation, migration and angiogenesis may be related to its targeted gene TRPM7.

In the present study, TRPM7 was identified as the target gene of miR‐9‐5p via luciferase assay. Accumulating evidence indicates that TRPM7 is essential for multiple cellular processes, including survival, proliferation and migration in stem cells and endothelial cells via PI3K/Akt pathways.^34^, ^35^ It has been well demonstrated that TRPM7 could inhibit the growth and migration of human umbilical vein endothelial cells (HUVECs).^36^, ^37^, ^38^, ^39^ Instead, inhibited or impaired the expression of TRPM7 could enhance the proliferation and migration, as well as angiogenesis of HUVECs, which might be due to the regulation of NO^36^ and the inhibition of autophagy.^40^

Hyun Geun Oh found that activation of the TRPM7 channel could increase Ca^2+^‐regulated basal autophagy.^41^ In addition, an inhibitor for TRPM7 channel decreased the level of basal autophagy.^42^ Besides, in the previous study, Zhang et al^40^ found that miR‐9 could inhibit autophagy via targeting Beclin1 3′UTR and thus enhances cisplatin sensitivity in A549 cells. A consistent result was found in EPCs in the present study.

In conclusion, we demonstrated that miR‐9‐5p promoted EPC proliferation, migration and angiogenesis via the mediated TRPM7 expression and PI3K/Akt/autophagy pathway.

## CONFLICT OF INTEREST

The authors declare that there are no potential conflicts of interest.

## AUTHOR CONTRIBUTIONS

Dong‐Ming Zhou and Li‐Li Sun: performed the laboratory work and the manuscript writing. Jian Zhu: performed the laboratory work and data analysis. Bin Chen: designed the research and drafted the manuscript. Wen‐Dong Li and Xiao‐Qiang Li: conception/design and fund raising, and final approval of the manuscript. All authors have reviewed and approved the final manuscript.

## Data Availability

The data used to support the findings in this study are available upon reasonable request from the corresponding authors.
